# To Better Generate Organoids, What Can We Learn From Teratomas?

**DOI:** 10.3389/fcell.2021.700482

**Published:** 2021-07-16

**Authors:** Hongyu Li, Lixiong Gao, Jinlin Du, Tianju Ma, Zi Ye, Zhaohui Li

**Affiliations:** Department of Ophthalmology, The Chinese People’s Liberation Army General Hospital, Beijing, China

**Keywords:** teratoma, organoid, 3D culture, ESC, IPSC

## Abstract

The genomic profile of animal models is not completely matched with the genomic profile of humans, and 2D cultures do not represent the cellular heterogeneity and tissue architecture found in tissues of their origin. Derived from 3D culture systems, organoids establish a crucial bridge between 2D cell cultures and *in vivo* animal models. Organoids have wide and promising applications in developmental research, disease modeling, drug screening, precision therapy, and regenerative medicine. However, current organoids represent only single or partial components of a tissue, which lack blood vessels, native microenvironment, communication with near tissues, and a continuous dorsal-ventral axis within 3D culture systems. Although efforts have been made to solve these problems, unfortunately, there is no ideal method. Teratoma, which has been frequently studied in pathological conditions, was recently discovered as a new *in vivo* model for developmental studies. In contrast to organoids, teratomas have vascularized 3D structures and regions of complex tissue-like organization. Studies have demonstrated that teratomas can be used to mimic multilineage human development, enrich specific somatic progenitor/stem cells, and even generate brain organoids. These results provide unique opportunities to promote our understanding of the vascularization and maturation of organoids. In this review, we first summarize the basic characteristics, applications, and limitations of both organoids and teratomas and further discuss the possibility that *in vivo* teratoma systems can be used to promote the vascularization and maturation of organoids within an *in vitro* 3D culture system.

## Introduction

The progress of clinical medicine cannot be separated from research on disease pathogenesis and drug screening. Restricted to ethical requirements, *in vivo* animal models and two-dimensional (2D) cell cultures have frequently been used to study human development and diseases in the last few decades. However, both models fail to mimic the complex architectures, cell-cell interactions, unique microenvironment and organ-level functionality of the human body (Kim et al., 2020). Since the isolation of human embryonic stem cells (ESCs) and the reprogramming of human somatic cells into induced pluripotent stem cells (iPSCs) succeeded, these pluripotent stem cells (PSCs) have become a source for cell replacement therapy and a new model to simulate human development (Hoffman and Carpenter, 2005). By exploring the self-organizing features of PSCs in a 3D culture environment, stem cell studies have improved our understanding of key aspects of organogenesis (Garreta et al., 2020). These advances have led to the generation of new cell culture systems named organoids that could be used to produce organ-like tissues. The first organoid was successfully generated from mouse ESCs and formed a fully stratified neural retinal tissue architecture in a spatiotemporal manner mimicking *in vivo* development (Eiraku et al., 2011). Then, a large number of culturing systems were introduced to generate a variety of tissue-specific organoids, including the brain, lung, intestine, liver, pancreas, and kidney (Lancaster et al., 2013; Watson et al., 2014; Dye et al., 2015; Broutier et al., 2016; Takasato et al., 2016). Organoids have compositions and architectures similar to living organs, which provides valuable information about the mechanisms underlying human development and organ regeneration. However, there are some limitations and challenges in current organoids, including the low reproducibility, the lack of blood vessels and native microenvironment, which limits their applications in disease modeling and drug screening (Fatehullah et al., 2016; Kaushik et al., 2018; Rossi et al., 2018). The development of the organoid system is still in its infancy in comparison to 2D cultures and animal models. Further study is instrumental and necessary to better generate and apply organoids.

In fact, there is a significant difference between *in vivo* and *in vitro* microenvironment. The microenvironment of 3D cultures is provided mainly by a solid extracellular matrix (ECM), which supports cell growth and promotes cell adhesion (Badekila et al., 2021). However, their complexity and variability in composition makes it more difficult to control the cultural microenvironment. Moreover, the characteristics of cell-to-cell adhesion and the other 3D properties are more sufficiently showed in *in vivo* than *in vitro* conditions (Aleckovic and Simón, 2008). And *in vivo* microenvironment is relatively stable and could not impede the infiltration of drugs comparing to a natural ECM with complex compositions.

Once PSCs grow in *in vivo* environment, teratomas would be formed. As a new *in vivo* model of human multilineage development, teratomas have similarities and differences with organoids (McDonald et al., 2020). Both organoid and teratoma are derived from PSCs. Organoid formation involves gradually controlled differentiation of PSCs and subsequent self-organization into tissue-specific organ-like structures. Teratoma formation involves uncontrolled differentiation and self-organization into various somatic tissues from all three germ layers. Teratoma systems provide an *in vivo* tool that enables more physiologically relevant experiments to be performed, which cannot be reproduced in animals or *in vitro* cultures. Recently, several studies showed that single specific lineage cell types, such as skeletal muscle stem cells, neural stem cells, and hematopoietic stem cells, have already been enriched from mouse PSCs through teratoma formation (Chan et al., 2018; Philipp et al., 2018; Kim et al., 2019). These enriched cells could be cultured *in vitro*, and even specific vascularized tissues could be isolated to generate organoids (Lee et al., 2020). Moreover, the variations of cell types in teratomas could be an advantage to generate complex tissues, which provides a possibility to investigate human development at a multi-organ level. All these results indicated that an *in vivo* teratoma system could provide a powerful platform to improve the method and technology of 3D culture. In this review, we substantially summarize the general characteristics, applications and limitations of current organoids and teratomas, and then focus on the progress and potential of teratoma systems as an *in vivo* tool for better generating organoids.

## Organoids: A Better *in vitro* Model

The 3D cultures have advantages over 2D cultures and animal models. When placed flat in a Petri dish, cells cannot behave as usual. Although animal models may be useful tools, they also have limitations because there are large differences between model animals and humans. Therefore, it is usually difficult to translate the results from animal models into physiological understanding of the human body with accuracy. The term “organoid” is defined as a complex 3D structure that develops from stem cells or organ-specific progenitors and displays architectures and functionalities similar to the architectures and functionalities of living organs (Ashok et al., 2020). Organoid systems are changing the way scientists model organ development and expanding basic biological research and medical research into a more physiologically meaningful human environment.

### Generating Organoids

#### Self-Organization of Organoids

The development of organoids usually involves the self-organization of a comparatively homogeneous cell population. Self-organization is the spontaneous formation of ordered patterns and structures from specific elements or individuals. The basic processes of self-organization involve the following three processes: self-assembly, self-patterning, and self-morphogenesis (Sasai, 2013). Self-assembly refers to the process of autonomously forming patterns by selectively gathering cells or rearranging the relative positions of cells (Whitesides and Grzybowski, 2002). Only through the self-assembly of one or several types of cells can highly ordered structures be formed (Dobrescu and Purcarea, 2011). Self-patterning is the spatial and temporal control of cell states to acquire heterogeneous properties in a region-specific manner (Sasai, 2013). Self-patterning starts with a symmetry disrupting event (Turner et al., 2016), which is affected by a variety of mechanisms, including reaction diffusion, asymmetric cell division, and stability of regulatory networks (Fleury and Watanabe, 2004; Sasai, 2013; Green and Sharpe, 2015). Self-morphogenesis, driven by internal organization mechanics and occurring automatically without external forces or spatial constraints, is the most crucial step that determines the final organoid formation (Sasai, 2013). Self-morphogenesis should include complex control of directionally internal forces and dynamic control of the cooperative response to mechanical force, as well as dynamic changes in tissue viscosity and stiffness (Lecaudey et al., 2008; Sasai, 2013). Successful formation of organoids depends on each described process, and three main features need to be carefully considered: the physical characteristics of the culture environment, the requirement for endogenous and exogenous signals, and the initial origin of culturing cell types (Rossi et al., 2018). The choice made for each of these features could affect the characteristics of the final organoid.

#### The 3D Culture Microenvironment

The microenvironment of 3D cultures is provided mainly by a solid extracellular matrix (ECM), which supports cell growth and promotes cell adhesion and is the most common way to enhance the 3D properties of organoids (Badekila et al., 2021). Matrigel, an animal-derived hydrogen from mouse sarcoma, is the initial natural ECM used in 3D cultures. Matrigel or another natural matrix (collagen I) is a complex mix of ECM components and growth factors, making cell growth and differentiation very efficient. However, their complexity and variability in composition makes it more difficult to control the cultural microenvironment and may reduce organoid-to-organoid reproducibility (Badekila et al., 2021). A hydrogel with chemical composition has recently been introduced to substitute for the undefined natural matrix (Gjorevski et al., 2016; Lindborg et al., 2016). Although hydrogels could control the mechanical and biochemical characteristics of the culture microenvironment, they need to be customized according to the specific requirements of different organoids because of their inherent low biological activity (Blondel and Lutolf, 2019). All these protocols derived for different specific organoids depend largely on experience (Rossi et al., 2018; Ashok et al., 2020). It is relatively difficult to evaluate the advantages and disadvantages of each protocol.

#### Endogenous and Exogenous Signals

The derivation of organoids relies partially/exclusively on endogenous or exogenous signals (Rossi et al., 2018). For example, mESC-derived optic cup organoids are generated under low growth factor conditions that support the formation of neuroepithelia, and then spatially separated domains of the neural retina and retinal pigmented epithelium are driven by self-organized mechanisms (Eiraku et al., 2011). The morphogenesis of neural retinal organoids proceeds without additional exogenous signals. However, in fact, most protocols for organoids require the addition of specific exogenous signals, as the starting cell population does not include all the necessary components to self-organize properly (McCracken et al., 2014; Takasato et al., 2014). The decision on which specific signals to use and when to apply them is generally obtained by the current understanding of relevant *in vivo* developmental mechanisms.

#### Starting Cell Type of Organoids

The characteristics of the final organoid also depend on the starting cell types. Organoids can be produced from PSCs, ASCs and fetal progenitor cells (Nakano et al., 2012; Fordham et al., 2013; Broutier et al., 2017). PSC-derived organoids have a complex composition containing epithelial, mesenchymal and endothelial cells. For instance, major cell populations of kidney organoids are tubular epithelia, mesenchyme and podocyte cells (Wu et al., 2018). However, not all differentiation processes of PSCs are efficient, and sometimes unexpected cell types may occur. Wu et al. (2018) found that in their kidney organoids, 10–20% of the cells are non-renal cells, including a neuronal lineage. Organoids derived from ASCs were first generated in the small intestine (Sato et al., 2009). ASC-derived organoids contain only the polarized epithelial components of organs and lack the stroma, nerves, and vasculature (Bartfeld et al., 2015; Huch et al., 2015). The ASC-derived organoids have lower complexity and predictability than PSC-derived organoids. The fundamental differences between PSC- and ASC-derived organoids make them complementary model systems. In addition, intestinal and pancreatic organoids could be derived from dissociated fetal cells or tissues (Fordham et al., 2013; Greggio et al., 2013).

### Applications of Current Organoids

The ability to grow near-physiological, self-renewing organoids provides us with a fabulous model system for basic research and transplantation application. Organoids are genetically stable, contain a variety of differentiated cell types, and produce specialized cell types that cannot be cultured *in vitro* (Schutgens and Clevers, 2020). Moreover, the self-organization of tissues and the spatiotemporal regulation of cells in organoids reproduce the development of human bodies. Following the establishment of human stem cell-derived organoids, various human diseases have been studied *in vitro*. Organoid systems have opened remarkable opportunities in human development and disease modeling, drug screening and precision therapy, and regenerative medicine (Fatehullah et al., 2016).

#### Development and Disease Modeling

As organoids retain the basic characteristics of their initial developmental stage, the detailed process of early embryonic development could be observed in a dish with the systematic induction of cell differentiation (Rookmaaker et al., 2015). For example, by inducing PSCs to differentiate into specific tissues, the involvement of FGF, BMP and Wnt signaling pathways in regulating the development of the brain, retina, stomach and pancreas has been clarified (Nakano et al., 2012; Greggio et al., 2013; Lancaster et al., 2013; McCracken et al., 2014). Additionally, a large number of disease models have been established with3D cultures, including infectious, genetic and tumor diseases, which provides a theoretical basis for reproducing the pathological features of human diseases (Qian et al., 2016; Dutta et al., 2017; Artegiani and Clevers, 2018; Drost and Clevers, 2018; Bresnahan et al., 2020; Campaner et al., 2020). For instance, *Helicobacter pylori* can be injected into stomach organoids to study the mechanisms of gastritis and the bacterial contribution to carcinogenesis (Salama et al., 2013). Patient-derived tissues or specific genetic mutations could be used to produce organoids for genetic or tumor disease modeling (Cristobal et al., 2017; Finnberg et al., 2017; Cruz and Freedman, 2018; Berkers et al., 2019; Kampmann, 2020; Teriyapirom et al., 2021). The combination of the CRISPR-Cas9 system and organoid technology would constitute a successful approach to investigate the underlying mechanisms and signaling pathways of genetic and cancer diseases through gene mutation, fusion and repair (Hendriks et al., 2020). A number of mutational combinations resulting from gene knockout based on the CRISPR-Cas9 system and gene insertion on transposons represent the diverse mutational patterns of brain cancer organoids (Bian et al., 2018). Similar applications were also found in intestine, liver and kidney organoids (Freedman et al., 2015; Drost et al., 2017; Artegiani et al., 2019). In summary, human organoids enable us to investigate in depth the processes that control embryonic development, present lineage characteristics, and mimic the occurrence and progression of diseases.

#### Drug Screening and Precision Therapy

There are often limitations, such as unpredictable results, time-consuming tests, and individual differences in newly developed drugs for human diseases (Eglen and Randle, 2015; Martin, 2015; Sampaziotis et al., 2015). The opinion that organoids could mimic the pathological process of human disease has provided a feasible tool for drug screening and precision therapy (Artegiani and Clevers, 2018). For example, models of colon cancers and cystic fibrosis could be replicated through a 3D culture system, and then corresponding biobanks could be established for drug testing (van de Wetering et al., 2015; Dekkers et al., 2016). A colon cancer biobank was used to screen 83 drugs currently applied as cancer treatment drugs in the clinic and confirmed the association of known genetic drugs (Bartfeld and Clevers, 2017). Similarly, organoids derived from cancer patients could be used to determine the ideal treatment for a particular patient because they preserve the genetic heterogeneity of the primary tumor (Huang et al., 2015; Nielsen et al., 2016). High-throughput screening paradigms are another advance in organoid-based drug screening, which was recently demonstrated in the kidney (Czerniecki et al., 2018). The generation of organoids based on specific diseases or even specific individuals is expected to become a powerful tool for precision therapy (Eglen and Randle, 2015; Walsh et al., 2016; Pasteuning-Vuhman et al., 2020).

#### Regenerative Medicine

Human organoids have also become a hopeful source of transplantable tissues and functional cell types in regenerative medicine. Proof-of-concept studies have already proven the transplantation of human organoids into animals (Takebe et al., 2015; van de Wetering et al., 2015). When intestinal organoids were transplanted into mice, colonic mucosa injuries regenerated (Cruz-Acuña et al., 2017). Organoids of neural retinas derived from mouse PSCs could be transplanted into a mouse with retinal degeneration (RD), which produced mature photoreceptor cells and constituted synaptic connections with the host cells (Assawachananont et al., 2014). Similar results existed in primate and rat models of RD (Shirai et al., 2016). In our previous study, we successfully used cell surface markers (C-Kit^+^/SSEA4^–^) to effectively eliminate tumorigenic embryonic cells and enriched retinal progenitor cells from human ESC-derived retinal organoids, which significantly improved vision and preserved retinal structure following subretinal transplantation into RD models of rats and mice (Zou et al., 2019). In addition, transplantation trials of kidney and liver organoids have also been successfully implemented in animal models (Taguchi et al., 2014; Huch et al., 2015). Although these results are preliminary, it is promising that cells and tissues derived from organoids might be a source for clinical transplantation.

### Limitations of Current Organoids

**FIGURE 1:**
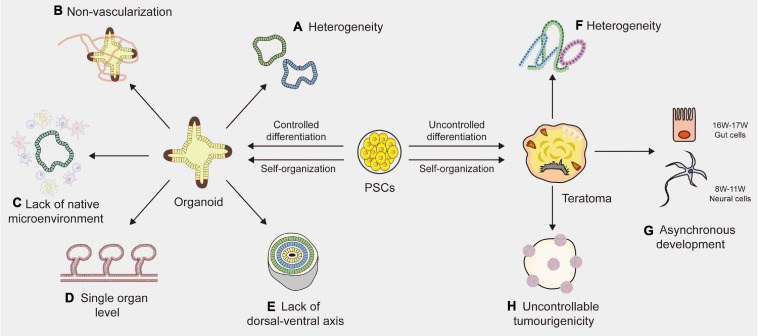
Limitations of organoid and teratoma. **(A)** Due to the lack of a reliable way to synchronize the size, shape and survival of organoids, the organoids vary among different laboratories. **(B)** The absence of blood vessels in organoids impedes their maturation and function. **(C)** Organoids lack native microenvironment which impede their applications in disease modeling and drug screening. **(D)** Most organoids are based on the single organ level and merely imitate a small part of the human body, not the entire part. **(E)** The absence of a continuous anterior-posterior or dorsal-ventral axis that guides and supports proper organoid directionality, especially for cerebral organoids. **(F)** Cell proliferation and differentiation are affected and controlled by cell lineages, host types and graft sites, resulting in the heterogeneity of teratomas. **(G)** The development of teratomas is asynchronous. In the 10-week teratoma, neural cells were highly similar to prefrontal cortex cells at human gestational weeks 16–17, while gut cells were most similar to human gut cells at gestational weeks 8–11. **(H)** The tumorigenicity of PSCs (pluripotent stem cells) is uncontrollable, leading to the uncontrollable tumorigenicity of teratomas.

As a new model that could substitute for *in vivo* research, organoids are growing faster in popularity than 2D cell culture and animal models because of their extreme fidelity. The application mentioned above depends on the repeatable formation of organoids that are highly similar to living organoids in terms of architecture and function (Rossi et al., 2018). Although the field of organoids is advancing at an impressive rate, there are some limitations and challenges that need to be resolved (Figures 1A–E).

#### Heterogeneity

One of the major limitations of human organoids is heterogeneity. Many protocols have been described to generate organoids, but there is still a lack of a widely accepted standardized solution. For example, intestinal organoids vary from different laboratories, where derivation from ASCs is exclusively composed of epithelial tissues, yet derivation from PSCs may generate mesenchymal tissues (Sato et al., 2011; Spence et al., 2011). Velasco et al. (2019) analyzed 166,242 cells isolated from 21 brain organoids using single-cell RNA sequencing and found that 95% of organoids produced almost indistinguishable cell types. Brain organoids generated by four distinct protocols showed significant differences in overall external morphology and size (Velasco et al., 2019). Due to the lack of a reliable way to synchronize the size, shape and survival of organoids, research design and data analysis become complicated, resulting in high variability of organoid phenotypes (Kaushik et al., 2018). Efforts should be made to develop clear guidelines for evaluating the quality and effectiveness of organoid systems. Although chemical hydrogel scaffolds and single-cell sequencing techniques are helpful to reduce the heterogeneity of organoids to some extent, generating a purified organoid with high tissue consistency remains an unsolved problem (Wang et al., 2017; Collin et al., 2019). A new standardized culture technique is needed to improve organoid-to-organoid reproducibility.

#### Non-vascularization

Another indispensable limitation is the absence of blood vessels, which impedes the maturation and function of organoids. Indeed, as the size increases to a specific level, nutrients and oxygen cannot fully reach the central part of the organoids, eventually resulting in cell death and growth arrest (Lancaster and Knoblich, 2014a,b). Vascular systems are specifically required for nutrient, oxygen and waste exchange, sometimes for signal transmission (Daniel and Cleaver, 2019). Moreover, blood vessels surrounding stem cells serve as a microenvironment for maintaining homeostasis, which plays a vital role in the differentiation and self-renewal of stem cells during embryonic development (Otsuki and Brand, 2017). The lack of vascular circulation systems can cause hypoxia and may accelerate necrosis during organoid culture, thus hindering the normal development and migration of cells (Fantin et al., 2013). To solve these limitations, studies have tried to produce vascularized organoids by co-culturing organoids with vascular endothelial cells or by combining organ-on-chip techniques with planting vascular endothelial cells (Takebe et al., 2013; Vatine et al., 2019). Yet most of these blood vessels are arranged in a disorderly manner and cannot replace the functionalities of living vessels (Vatine et al., 2019; Shi et al., 2020). Transplanting organoids into immunodeficient mice is a simple way to generate blood vessels (Mansour et al., 2018). However, it is difficult to estimate the source of vascular endothelial cells because whether organoids contain host- or graft-derived blood vessels relies on the location of transplantation (Daniel and Cleaver, 2019). Currently, a functional vascular-like system has been established in human brain organoids, by expressing ETS variant 2 (ETV2) that contributed to forming a complex vascular-like network in cortical organoids (Cakir et al., 2019). Another stable and reproducible method is needed to establish vascularized organoids of the others. As vascular endothelial cells display stark heterogeneity based on their local environment, there is a long way to go before achieving the vascularization of organoids (Daniel and Cleaver, 2019).

#### Lack of Native Microenvironment

The third limitation of organoids is the lack of native microenvironment that precludes studies about interaction of stem cells with their niches or immune cells, etc., particularly in adult stem cell-derived organoids (Fatehullah et al., 2016; Drost and Clevers, 2018; Ashok et al., 2020; Kim et al., 2020). The modeling of cell-to-cell communication with immune systems and the development of vascular networks in organoid systems are essential to be addressed. Significant progress has recently been achieved with co-culture systems, such as tumor organoids with immune cells (Nozaki et al., 2016; Finnberg et al., 2017). Yet these immune cell populations are less mature, or could only be maintained for a short period of time that is not conducive to investigating the long-term response of immune systems to chronic diseases or new drugs (Drost and Clevers, 2018). Besides, most of human organoids do not robustly retain the complex full diversity and physical architecture of the native microenvironment (Neal et al., 2018). Controlling the culture environments by engineering approaches may supply repeatable experimental conditions and results (Hagiwara and Koh, 2020). In addition, it is possible to quantitatively change the environmental condition by controlling the microenvironment surrounding the cells (Rossi et al., 2018; Hagiwara and Koh, 2020). However, it still remains a difficult obstacle to overcome.

#### Other Limitations

Other than the limitations described above, human organoids still face many challenges. First, current organoids merely imitate a small part of the human body, not the entire part, because only organoids of a single organ origin have been established (Fatehullah et al., 2016). Human organoids still lack communication with near organs or tissues and are limited to studying the reproduction of organ-specific or tissue-specific microphysiology (Kim et al., 2020). A multichannel 3D microfluidic system or a chamber device, which enables the individual cultivation of different organoids, may facilitate organoid-to-organoid communication in the future (Zhang et al., 2009; Achberger et al., 2019; Koike et al., 2019). However, there is a long way to go before implementing the human multiorganoid model *in vitro*. Additionally, the lack of a dorsal-ventral axis is another issue which affects the proper directionality of cerebral organoids (Ashok et al., 2020). Although some efforts are in progress to improve these limitations, current organoids still lack functions, such as light responsiveness of retinal organoids (Lancaster et al., 2013), and filtering blood of kidney organoids (Homan et al., 2019).

All of the abovementioned methods to solve these limitations rely heavily on an *in vitro* environment that provides essential materials for cell growth through the addition of growth factors. The *in vitro* environment is unable to provide blood vessels, intercellular junctions and specific factors, which develop in *in vivo* conditions. A new technique developed under *in vivo* conditions might be an opportunity to address these challenges. Human PSC-derived teratomas have the same cellular origin as human organoids and a similar self-organizing pattern. Researchers indicated that, as teratomas could generate a wide array of cell types and grow to a large size because of their vascularization, they could be used as a model for multilineage human development (McDonald et al., 2020). In view of the rapid technical development in this field, we believe that teratomas are in a time of extraordinary opportunity to better generate organoids (Suzuki et al., 2013; Lee et al., 2020; McDonald et al., 2020).

## Teratomas: A Emerging *in vivo* Model

In recent years, studies of human embryonic development have been limited by a scarcity of relevant biological material and key ethical constraints. Although organoids have solved these limitations to a certain extent, they can only be studied in an *in vitro* setting. Thus, there has been a push to establish *in vivo* models specific to human development. Teratomas are derived from PSCs and formed under *in vivo* conditions. As teratomas contain tissues from all three embryonic germ layers, they have become an emerging *in vivo* model in stem cell research (Thomson et al., 1998). In this section, we mainly discuss the general characteristics of teratomas and their applications and limitations.

### General Characteristics of Teratomas

The term “teratoma” is derived from the Greek words “teras” meaning monster and a suffix denoting a tumor (Damjanov and Andrews, 2007). Teratomas are deemed tumors due to their progressive, uncoordinated and unregulated growth. The term has been used to describe both benign and malignant tumors composed of multiple tissues foreign to the anatomic site from which they originate. Currently, researchers use the term as simply “teratoma” for benign tumors and “teratocarcinoma” as malignant tumors. Natural teratomas in humans are benign germ cell tumors that occur mainly in gonads, such as ovaries and testes (Damjanov and Solter, 1974). A teratoma could occasionally be found in the retroperitoneum and anterior mediastinum. Experimental teratomas are generated by transplanting PSCs into immunodeficient mice, which is the most essential approach to assess the differentiation potential of cells (Lensch et al., 2007). Both kinds of teratomas are composed of various somatic tissues arranged in a random manner and can form in humans and animals (rats and mice) (Bulic-Jakus et al., 2016). Histologically, teratomas contain highly organized structures on behalf of all three embryonic germ layers: nerve and epidermis tissue from ectoderm, bone and muscle tissue from mesoderm, bronchus and gut tissues from endoderm (Thomson et al., 1998). In this section, we systematically expound the characteristics of experimental teratomas.

Experimental teratomas are generated by transplanting normal PSCs to an ectopic site (Hultman et al., 2014). The *in vivo* environment of teratoma system could provide necessary growth factors and critical signaling pathways, to promote cell growth and differentiation. It also has a more sufficient cell-to-cell adhesion and 3D properties than *in vitro* microenvironment, which is relatively stable and could not impede the infiltration of drugs comparing to a natural ECM with complex compositions (Aleckovic and Simón, 2008). Yet it is worth noting that the *in vivo* environment might modify tumorigenesis of stem cells, through specific epigenetic pathways (Berger et al., 2009) or by eliciting an immune response (Bulic-Jakus et al., 2016). The environmental cues within the embryo influence the proliferation and differentiation of hESC after transplantation (Cunningham et al., 2012). The normal PSC spontaneously organizes when PSCs are injected into immunodeficient mice by subcutaneous, intramuscular, or intratesticular routes (Hentze et al., 2009). Teratomas are strongly dependent on the site of engraftment, and the simplest and most effective method is subcutaneous implantation. Generally, at least 10^3^ PSCs must be transplanted to guarantee the formation of teratomas (Thomson et al., 1998; Heins et al., 2004). However, no evidence has indicated that the quantity of implanted cells has an effect on teratoma formation. In addition, teratomas are generally surrounded by a capsule that prevents integration of stem cells into host tissues and limits cell-cell interactions. Cells with teratomas are exactly mature, easy to remove and do not invade adjacent tissues (Gertow et al., 2007). Although the tissue arrangement in teratomas seems to be chaotic, their proliferation and differentiation are not completely random but are affected and controlled by many factors, such as ESC lineages, host types and graft sites (Aleckovic and Simón, 2008). Since human ESC lineages are derived from individual embryos, they may have different differentiation potency, even in monozygotic twin ESC lines (Lauss et al., 2005). Studies have demonstrated that the level of mRNA expression differs among different human ESC lineages (Skottman et al., 2005; McDonald et al., 2020). The structure of teratomas derived from different hESC lineages may also exhibit differences to a certain extent. Previous studies have shown that the differentiation of ESCs is affected by neighboring cells and signaling molecules from the microenvironment (Blum and Benvenisty, 2007). For instance, ESCs injected subcutaneously have a higher incidence of teratomas, teratomas in the knee grow slower and smaller (Wakitani et al., 2003), and teratomas in the liver grow faster and contain many fluid-filled cavities (Cooke et al., 2006). Current research on the detailed characteristics of differentiation events after human PSC xenotransplantation is still limited (Hultman et al., 2014). However, most of the human PSC xenotransplantation events focus mainly on basic morphological visualization and lack a consensus standard protocol to generate experimental teratomas, which requires further exploration (Gropp et al., 2012).

### Applications of Teratomas

Teratomas provide a new model for stem cell research. The *in vivo* growth environment indicates that the teratoma system can highly reproduce the differentiation of PSCs and embryonic development in the early stage under physiological conditions. In addition to being used for pluripotency testing, the teratoma system has also shown potential applications in human development modeling, disease modeling, and tissue engineering.

#### Tumorigenicity and Pluripotent Assay

Experimental teratomas were primitively a tumor test before they were used to prove the pluripotency of stem cells (Peterson et al., 2011). As human stem cell therapies have been available for diseases and injuries, such as diabetes, macular degeneration, cancer, and spinal cord injury in recent years, tumorigenicity assays are especially important (Yamanaka, 2020). Only non-tumorigenic stem cells can be safely injected into the human body for treatment. Due to the low immunity of immunodeficient animals, the short lifespan of rodents, or the requirement of specific factors to support tumor growth, teratoma assays may underestimate tumorigenic potential, which impedes long-term monitoring of transplanted stem cells (Cunningham et al., 2012). Another important application of teratomas is to test whether cells are pluripotent (Knoepfler, 2009; Ben-David and Benvenisty, 2011; Cunningham et al., 2012; Buta et al., 2013). Pluripotent assays are indispensable for identifying the multidirectional differentiation potential of PSCs, and experimental teratomas are the most common and simple method to verify the pluripotency of isolated ESCs and reprogrammed iPSCs. However, there is insufficient proof to fully demonstrate the composition and benignancy of experimental teratomas (Hultman et al., 2014). To better detect tumorigenicity and pluripotency with pertinence, the standardization of teratoma assays should be an essential goal that has not yet been achieved (Reubinoff et al., 2000).

#### Development and Disease Modeling

Teratomas could provide information about the molecular pathways and mechanisms in early embryonic development by patterning developmental processes (Aleckovic and Simón, 2008). A “developmental gradient” will form within the teratoma during its development: undifferentiated stem cells in the center, followed by tissues in the early developmental stage and tissues in the late developmental stage in the outermost layers (Wernig et al., 2007). This different developmental gradient might be useful to assess the gradual differentiation process and related molecular mechanisms of stem cells. Recently, McDonald et al. (2020) performed single-cell RNA sequencing of 179,632 cells across 23 teratomas from three human ESC lines and one human iPSC line and found that teratomas reproducibly contained approximately 20 cell types. Every teratoma cell type is highly associated with at least one mouse fetal cell type (McDonald et al., 2020). McDonald et al. (2020) also used the CRISPR-Cas9 system to establish a single-cell genetic knockout screen on 24 main lineage-specific genes and to assay the functions of multiple lineage genes that are critical to human development, proving that teratomas were able to act as new models for studying multilineage human development, pantissue functional genetic screening, and tissue engineering (Aleckovic and Simón, 2008).

Moreover, disease models could be successfully established by teratomas to investigate pathogenic genes and regulatory mechanisms (Tzukerman et al., 2003; McDonald et al., 2020). In McDonald’s study, three rare neurodevelopmental diseases, including Rett, L1, and Pitt-Hopkins syndromes, were built combining genetic knockout systems and teratoma formation (McDonald et al., 2020). Additionally, teratoma formation could also be applied in cancer research (Hultman et al., 2014). When ovarian tumor cells were transplanted into mature teratomas of immunodeficient mice, tumor cells invaded into surrounding normal differentiated tissues and led to the growth of new blood vessels (Tzukerman et al., 2003). Therefore, teratomas might serve as an adequate experimental model to study and manipulate the local microenvironment in the growth of tumor cells, thereby contributing to the progress of cancer research. In general, the teratoma system provides a new platform to deepen our understanding of human development and better establish disease models (Menendez et al., 2006).

#### Tissue Engineering

Furthermore, the complex organ-like structures of teratomas may provide a new approach to study tissue engineering (Aleckovic and Simón, 2008). Current tissue engineering attempts to imitate the *in vivo* environment to produce a more realistic *in vitro* cell differentiation model (Zheng et al., 2018). However, due to the multidirectional differentiation potential of stem cells, it is difficult to control their arrangement into a high-order structure composed of multiple cell types (Hoffman and Carpenter, 2005). Unlike *in vitro* models, experimental teratomas could differentiate into multiple lineage tissues *in vivo*, which reproduces the differentiation process of living cells in high fidelity (Buta et al., 2013). Finding the possible process and mechanism of the complex organ-like structure in the teratoma can increase the knowledge of tissue growth and may be helpful to generate functional tissues. Overall, experimental teratomas may not only represent proof of pluripotency but can also be used in several areas, such as embryogenesis, cancer research, tissue engineering and regeneration medicine.

### Limitations of Current Teratomas

Although the teratoma system has demonstrated its potential as a new *in vivo* model, there are some limitations (Figures 1F–H). One of the important limitations is heterogeneity (Velasco et al., 2019). Previous study demonstrated that tissue arrangement in teratomas and their proliferation and differentiation were affected and controlled by stem cell types, nature of the PSC line, nature of the host and graft sites (Aleckovic and Simón, 2008). All these factors, especially different stem cell lines, would affect the structures and components of the final teratoma. To control heterogeneity, the standardization of generation methods is important (Müller et al., 2010). The most effective way is to transplant the same PSC line in the same site from the same host type. Additionally, genetic engineering technology is also a useful method to minimize the variations of teratoma. For instance, through miRNA-based molecular sculpting, teratomas could be engineered toward a desired lineage to apply for studying developmental biology and human disease (McDonald et al., 2020). Moreover, desired cell types could be enriched in teratomas by genetic engineering to investigate tissues of our interest (Suzuki et al., 2013; Lee et al., 2020). Another limitation of teratomas is their complicated somatic tissues arranged in a semirandom manner. Indeed, the tumorigenicity of PSCs is uncontrollable (Singh et al., 2016). A previous study showed that the genetic and epigenetic differences between ESCs and iPSCs have an effect on their tumorigenicity (Ben-David and Benvenisty, 2011). When transplanting human fetal grafts into severe combined immunodeficient mice, undifferentiated tumors could be generated (Shih et al., 2007). Autologous iPSCs that present no immune barrier would increase the chance of generating a teratoma (Kawamata et al., 2015). Sometimes teratomas even contain organotypic tissues (e.g., hair, teeth, limbs), resulting in difficulty in studying the properties of the tissues of interest (Bulic-Jakus et al., 2016). The third limitation is the asynchronous development of teratomas. A study indicated that in the 10-week teratoma, neural cells were highly similar to prefrontal cortex cells at human gestational weeks 16–17, while gut cells were most similar to human gut cells at gestational weeks 8–11 (McDonald et al., 2020). The impact of this asynchronous development was two-pronged. The upside is that tissues in the late stage of human development could be generated through short-term culture of the tissues isolated from teratomas. The downside is that this asynchronous manner can become an obstacle to capture specific mature cell types developed in a highly ordered microenvironment. Therefore, it is urgent to find a unique dissociation protocol that could capture cell types as specific as possible. In addition, teratomas were generally induced in mice and rats that had a relatively short lifespan, which impeded the long-term observation of diseases that may persist in humans for several years (Cunningham et al., 2012). Further studies on teratoma systems are significantly needed to overcome these limitations.

## Lessons Form Teraomtas to Better Generate Organoids

**FIGURE 2:**
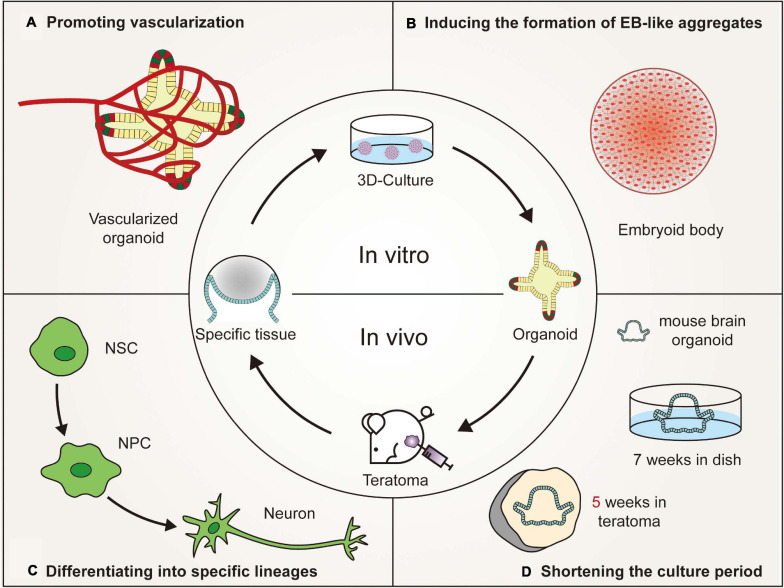
Advantages of *in vivo* teratoma systems to better generate organoids. **(A)** Vascularized tissues could be isolated from *in vivo* teratomas by genetic engineering and then cultured *in vitro* to generate vascularized organoids. **(B)** Pluripotent stem cells (PSCs), showing poor embryoid-body (EB)-forming ability *in vitro*, are usually defective in the initial stage of differentiation. However, the PSCs could form teratomas *in vivo* to generate embryoid-body-like aggregates to promote organoid formation. **(C)** By combining teratoma systems with genetic engineering technology, teratoma systems could be used to enrich specific lineage cells with more accuracy to better generate organoids and improve the reproducibility of organoids. **(D)** Mouse embryonic stem cell-derived brain organoids, which are produced in 7 weeks by *in vivo* 3D culture, could be generated in 5 weeks by *in vitro* culturing nerve tissues isolated from teratomas (NSC, neural stem cell; NPC, neural progenitor cell).

The use of organoid platforms has led to advancements in *in vitro* organogenesis and disease modeling. However, the variations between organoids and living organs seriously hinder the applications of organoids. Teratoma systems provide a more advanced *in vivo* tool that enables more physiologically relevant experiments to be performed, which cannot be reproduced in animals or *in vitro* cultures (Figure 2). Teratomas provide a microenvironment that is more suitable for stem cell growth. The teratoma system facilitates the formation of embryoid-body-like aggregates from stem cells with poor ability to differentiate and could also be used to enrich specific cell lineages (Lee et al., 2020). Moreover, teratomas spontaneously develop blood vessels with a short duration of neoplasia, which could promote vascularization and shorten the cultivation time of organoids (Stachelscheid et al., 2013; Lee et al., 2020; McDonald et al., 2020).

### Inducing the Formation of Embryoid-Body-Like Aggregates

Embryoid bodies (EBs) usually refer to the 3D structure spontaneously formed during the suspension culture of PSCs (Itskovitz-Eldor et al., 2000). Similar embryoid-body-like aggregates are also formed in the early developmental stage of 3D culture, which requires the involvement of specific inducible factors (Watanabe et al., 2005; Eiraku et al., 2008; Sasai, 2013). The formation of embryoid-body-like aggregates is the first step in organoid formation. However, partially reprogrammed iPSCs sometimes fail to differentiate *in vitro*, resulting in the inability of an embryoid-body-like aggregate to form (Lee et al., 2020), possibly because the isolated iPSCs retain some somatic memory, at least at low passages (Kim et al., 2011; Ohi et al., 2011). The other reason for the failure of an embryoid-body-like aggregate to form is that the established iPSC lines may contain aberrant traits, which may be caused by the stoichiometry of reprogramming factors and culture conditions (Newman and Cooper, 2010; Carey et al., 2011). Moreover, appropriate differentiation protocols should be applied to induce *in vitro* differentiation of PSCs, depending on the intrinsic trait of differentiation potency of each cell line, especially in iPSCs (Liang and Zhang, 2013). Induced PSCs have differential identities distinct from ESCs, attributing to the different epigenetic regulatory mechanisms which play important roles in shaping the cellular identity (Bock et al., 2011). The different characteristics of iPSCs have been suggested to simply reflect the polymorphism of pluripotency that can be observed in variable ESC lines (Guenther et al., 2010; Newman and Cooper, 2010). A more effective method is required to ensure that PSCs can form embryoid-body-like aggregates to promote organoid formation.

Unlike the formation of EBs, teratomas are relatively easy to implement (Lee et al., 2009). Histologically, EBs have a much lower level of differentiation than teratomas. The central area of EBs may be necrotic (Stachelscheid et al., 2013). However, teratomas are completely differentiated and composed mainly of neuroectodermal and mesenchymal tissues (McDonald et al., 2020). Differentiation through teratoma formation could be universally applicable because not only naive PSCs but also partially reprogrammed cells could form teratomas (Hong et al., 2016; Kim et al., 2017). Lee et al. (2020) showed that neural differentiation through teratoma formation presented similar results irrespective of the cell type used. Embryoid-body-like aggregates that are difficult to form *in vitro* could be induced by *in vivo* teratoma formation and then through suspension culture or other *in vitro* culture methods to promote organoid formation. Moreover, PSC-derived tissues that have developed to a certain stage *in vitro* could be transplanted subcutaneously into mice and differentiated according to teratoma development patterns, or related growth factors can be added to promote organoid formation. Due to the limited number of correlative studies, more studies focusing on this aspect can be carried out in the future.

### Differentiating Into Specific Lineages

Under 3D culture conditions, PSCs first differentiated into progenitor cells and subsequently formed tissue-specific organoids (Foley, 2017). However, these organoids commonly include multiple lineage cell types (Lancaster and Knoblich, 2014a). For instance, single-cell sequencing results showed that kidney organoids contained non-renal cells, including three neuronal clusters and one muscle cluster, and that retinal organoids contained fibroblast cells and vascular cells (Wu et al., 2018; Wang et al., 2021). If progenitors from a single lineage could be enriched *in vitro*, the production of unexpected organoid cells could be reduced, and the target organoid would be relatively easy to obtain. In recent years, several studies have successfully enriched single lineage progenitor/stem cells through a teratoma formation system (Amabile et al., 2013; Hong et al., 2016; Chan et al., 2018). As early as 2013, Suzuki et al. (2013) found that hematopoietic stem niche-like cells were present in iPSC-derived teratomas and even migrated into the bone marrow of mice. When these cells were injected intravenously into irradiated recipients, lymphoid and myeloid cells could be reconstituted (Amabile et al., 2013). Moreover, injecting Olig2-GFP transgenic ESCs into immunodeficient mice could also generate neural stem cells that then differentiated into terminal neuronal and glial cells (Hong et al., 2016). Neural stem cells could also be obtained in the primary culture of human infantile teratomas (Kim et al., 2019). In addition, Chan et al. (2018) showed that myogenic progenitors could be produced from mouse PSCs without genetic modification through teratomas. Only 40,000 myogenic progenitor cells transplanted into diseased muscles of NSG-mdx(4Cv) mice regenerated 80% of the total muscle volume, which matured into functional muscle cells *in vivo* and was able to improve force generation (Chan et al., 2018).

However, the semirandom differentiation trait of teratomas makes it difficult to distinguish various tissues, as different cell lines might be found in the same spatial region (Bulic-Jakus et al., 2016). McDonald and his colleagues applied genetic engineering techniques to solve this issue at the single-cell level, which they called the miRNA circuit technique (McDonald et al., 2020). Neural lineage cells, including early neurons, neuronal progenitors and Schwann cells, could be enriched by regulating tissue-specific miRNA-124. Furthermore, clumps of enriched neural lineage cells through teratoma formation might be cultured *in vitro* to generate brain organoids, which had a better consistency in terms of cellular arrangements and tissue elements compared to traditional 3D culture systems (Lee et al., 2020). Normally, PSC-derived teratomas are well-differentiated (Lensch et al., 2007). By combining genetic engineering technology, a teratoma system could be used to enrich cells from specific lineages with more accuracy (McDonald et al., 2020), which is a good example of reducing heterogeneity and improving the reproducibility of current organoids.

### Promoting Vascularization

Vasculogenesis refers to the process by which capillaries sprout from existing blood vessels and invade avascular tissues (Risau, 1997). On the 18th day of human embryo development, endothelial cells can be differentiated from the endoderm and subsequently form vascular networks that invade embryonic tissues (Risau and Flamme, 1995). These vascular networks eventually differentiate into arteries, veins, and lymphatic tissues (Semenza, 2007). The development of human organs and blood vessels is synchronous (Ruhrberg et al., 2002). Vascular systems provide nutrients and oxygen to promote maturation and maintain the normal function of human organs. However, in the 3D culture environment, blood vessels cannot follow the formation of organoids (Eiraku et al., 2008; Nakano et al., 2012), which limits both the size enlargement and functional maturity of organoids (Kim et al., 2020). Although several technologies, such as co-culture, 3D printing, and bioreactors have already been adopted to promote the vasculogenesis of organoids, the volume of organoids is still much smaller than the volume of embryonic organs (Takebe et al., 2013; Phelan et al., 2019; Brassard et al., 2020). *In vitro*-generated blood vessels are arranged in a disorderly manner, and there is a lack of blood supply to verify their functions to transport oxygen and nutrients (Ashok et al., 2020).

However, human PSC-derived teratomas offer a complex 3D microenvironment, with corresponding tissue patterning, extracellular matrices and epigenetic imprinting, to support vessel development (Gertow et al., 2004; Damjanov and Andrews, 2016). Little is known about the formation and maintenance of blood vessels in teratomas. Some studies have suggested that the hematopoietic cell phenotype may reside in bone marrow-like structures, liver cells and mesenchymal stroma of teratomas (Amabile et al., 2013; Dekkers et al., 2016). Vascularized tissues could be isolated from *in vivo* teratomas by genetic engineering or obtained from *in vitro* teratomas cultured in a 3D perfusion bioreactor (Stachelscheid et al., 2013; Lee et al., 2020; McDonald et al., 2020). Then, these isolated specific vascularized tissues could be cultured *in vitro* to generate vascularized organoids. However, due to the small number and disordered arrangement of blood vessels in teratomas, it is relatively difficult to generate vascularized organoids, such as the brain and kidney that require a plentiful blood supply (Gerecht-Nir et al., 2004). Recently, Wimmer et al. (2019) reported the development of self-organizing 3D blood vessel organoids from PSCs, which produced a stable perfused vascular tree after transplantation under the renal capsule of mice. Blood vessel organoids could be transplanted into the site of teratoma formation or co-cultured with isolated specific vascularized tissues to increase the number of blood vessels. Moreover, organoids could be transplanted into mice to promote organoid vascularization according to the development pattern of teratoma vascular systems. Hematopoietic stem cells or vascular endothelial cells could be coinjected into mice to promote vascular formation (Philipp et al., 2018; Wimmer et al., 2019). In brief, the vascular system in teratomas provides a reliable platform for organoid vascularization (Gerecht-Nir et al., 2004).

### Shortening the Culture Period

The culture time of organoids varies according to different organ types (Spence et al., 2011; Nakano et al., 2012; Lancaster et al., 2013; van den Berg et al., 2018). In general, the more precise and more complex the tissue, the longer it takes to grow. With increasing culture time, the center of the medium gradually lacked the exchange of nutrients and oxygen (Lancaster and Knoblich, 2014a,b), resulting in retinal organoids dying in 126 days (Nakano et al., 2012). Brain organoids also start to shrink after 6 months (Lancaster et al., 2013). The maximum diameter of organoids is only 4 mm (Lancaster et al., 2013). Current organoids are similar to fetal tissues in the early stage of development (Fatehullah et al., 2016; Ashok et al., 2020). To obtain mature organoids, one method is to promote vascularization (Rossi et al., 2018), and the other is to shorten the cultivation time, which theoretically might speed up the differentiation of PSCs before nutrients run out in a limited space, prolonging the lifespan of organoids (Lee et al., 2020).

One advantage of using the teratoma system to generate organoids is that using the teratoma system can shorten the time of 3D culture. Generally, when 10^6^ undifferentiated diploid mouse ESCs are inoculated into immunodeficient mice, teratomas arise in a high proportion of recipients within 3–6 weeks (Gertow et al., 2004; Cooke et al., 2006; Valbuena et al., 2006; Hentze et al., 2009; Prokhorova et al., 2009). An mESC-derived brain organoid was generated in 5 weeks by *in vitro* culturing nerve tissues isolated from a teratoma (Lee et al., 2020). Compared with traditional 3D culture, the cultivation time of mouse brain organoids was reduced by nearly 2 weeks (Eiraku et al., 2008). In the traditional 3D culture microenvironment, PSCs are involved in stepwise differentiation and subsequent self-organization, mimicking tissue formation and organogenesis in mammalian embryonic development (Morgani et al., 2017). These processes could be partially generalized in teratoma formation, although they involve uncontrolled differentiation and self-organization (Singh et al., 2016). The characteristic of uncontrolled differentiation may be related to the asynchronous development of teratomas. Usually, the closer to the center of the teratoma, the later the tissue develops, which is determined by the developmental gradient of the teratoma (Wernig et al., 2007). For example, nerve cells develop earlier than embryonic development, and gut cells develop later than embryonic development (McDonald et al., 2020). Therefore, tissues that developed faster than embryonic development could be isolated from teratomas and cultured *in vitro* to shorten the culture time of the corresponding organoids. However, considering that teratoma formation from human PSCs takes twice as long (nearly 2–3 months) as teratoma formation from mouse PSCs, this method is more suitable for mice than humans to shorten the time of organoid formation (Lee et al., 2020). Future studies must focus on promoting the maturity of human-derived teratomas and the maturity of tissues that develop later than embryonic development in teratoma formation.

## Conclusion and Future Perspectives

In conclusion, the development of human organoids is in the primary stages, and there are many limitations impeding their applications. The teratoma system, as an emerging *in vivo* model, provides a potentially encouraging opportunity to better generate human organoids: inducing the formation of embryoid-body-like aggregates that are difficult to produce *in vitro*; differentiating into specific progenitor cell lineages that might increase organoid-to-organoid reproducibility; promoting vascularization that would prolong the organoid lifespan to create mature functional 3D tissues; and shortening the culture period of organoids by teratoma formation and 3D culture binding methods.

To induce the differentiation of PSCs *in vitro*, appropriate differentiation protocols should be applied according to the characteristics of each cell line because each cell line has different intrinsic differentiation potential, especially in iPSCs (Liang and Zhang, 2013). However, when PSCs are transplanted into mice, they spontaneously form a vascularized and highly organized 3D structure without any additional intervention. The teratoma system provides specific *in vivo* environmental factors that promote the maturation of organoids. The 3D tissues grown *in vitro* to a certain stage (embryoid-body-like aggregates) could be transplanted subcutaneously into mice to promote blood vessel formation according to the development pattern of teratomas. The 3D tissues could also be coinjected with vascular endothelial cells, hematopoietic stem cells and even vascular organoids (Philipp et al., 2018; Wimmer et al., 2019). Additionally, specific tissues could be isolated from teratomas using genetic engineering techniques and further cultured *in vitro* to obtain a single lineage organoid. The *in vivo* and *in vitro* conjugation method is a potential tool to better generate organoids. Future studies should be conducted to investigate the possibility of these hypotheses. Moreover, a series of new technologies, such as co-culture, organ-on-chip, and CRISPR-Cas9-mediated gene editing that have been used in organoids could also be applied to the teratoma system to better generate organoids (Koledova and Lu, 2017; Takebe et al., 2017; Hendriks et al., 2020). In the near future, the potential of the *in vivo* teratoma system, coupled with advances in organoids, is expected to provide a series of powerful and efficient platforms for studying human development, physiology and disease.

## Author Contributions

HL wrote the manuscript. HL and LG generated the figures. LG, ZY, and ZL revised the manuscript. All authors contributed to the article and approved the submitted version.

## Conflict of Interest

The authors declare that the research was conducted in the absence of any commercial or financial relationships that could be construed as a potential conflict of interest.
